# Hesperetin attenuates LPS‐induced the inflammatory response and apoptosis of H9c2 by activating the AMPK/P53 signaling pathway

**DOI:** 10.1002/iid3.973

**Published:** 2023-08-10

**Authors:** Zan Xie, Chunxia Yang, Tingting Xu

**Affiliations:** ^1^ Department of Cardiology the Affiliated Yantai Yuhuangding Hospital of Qingdao University Yantai Shandong China

**Keywords:** AMPK, apoptosis, hesperetin, inflammation, lipopolysaccharide, P53

## Abstract

**Introduction:**

Hesperetin (HES), whose main pharmacological effects are anti‑inflammatory and cardioprotective properties. In our study, we investigated the role of HES in lipopolysaccharide (LPS)‐induced inflammation and apoptosis in H9c2 cells.

**Methods:**

Cell viability was assessed through MTT assay. Tumor necrosis factor (TNF)‐α and interleukin (IL)‐β expression were quantified through RT‐qPCR assay. Secondly, the apoptosis rate was assessed by Terminal deoxynucleotidyl transferase‐mediated dUTP nick end labeling assay. Finally, B‐cell lymphoma 2 (Bcl‐2)‐ associated X protein (Bax), adenosine monophosphate‐activated protein kinase (AMPK), and P53 expression were quantified through western blot assay.

**Results:**

Our results demonstrated that LPS stimulation decreased the cell viability, increased IL‐1β and TNF‐α expression in H9c2 cells. However, HES treatment significantly increased the cell viability, decreased IL‐1β and TNF‐α expression in LPS‐induced H9c2 cells. In addition, HES significantly increased the phosphorylation level of AMPK. Meanwhile, HES prevented against LPS‐mediated the P53 and Bax protein upregulation, and Bcl‐2 protein downregulation in H9c2 cells. More interestingly, compound C (an AMPK inhibitor) treatment eliminated the protective effects of HES.

**Conclusion:**

Our findings revealed that HES attenuated the LPS‐mediated inflammation and apoptosis of H9c2 cells by activating the AMPK/P53 signaling pathway, suggesting that HES may be a potential cardioprotective agent.

## Graphical abstract

Our findings demonstrated that HES attenuated the LPS‐induced inflammation and apoptosis of H9c2 cells by activating the AMPK/P53 signaling pathway, suggesting that HES may be a potential cardioprotective agent.

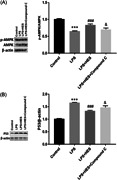

## 1 INTRODUCTION

The pathogenesis of cardiovascular disease is complex, and myocardial disease is one of the diseases with high incidence in cardiovascular disease.^1^, ^2^ Accumulating evidence demonstrates that cardiomyocytes' apoptosis and inflammatory responses are key processes in cardiomyopathy. Moreover, inflammation and apoptosis of cardiomyocytes are also prominent pathological features in other cardiovascular diseases such as sepsis, nerve damage, and myocardial infarction injury.^3^, ^4^, ^5^, ^6^ While traditional therapies to prevent cardiomyocyte inflammation and apoptosis remain ineffective, research has focused on new strategies.^7^, ^8^, ^9^, ^10^, ^11^

Lipopolysaccharide (LPS) is involved in regulating the initiation of pathophysiological cascades.^12^ Previous literature reported that reduction of LPS could improve outcomes in patients with cardiac disease.^13^ LPS treatment of H9c2 cells can lead to an inflammatory response.^14^ H9c2 cardiomyocytes have also been used to construct disease models, providing a new scientific approach to the treatment of chronic heart failure.

Hesperetin (HES) is an active ingredient from flowers, with pharmacological activities such as anti‐inflammatory, neuroprotective, and cardioprotective.^15^, ^16^, ^17^ In addition, HES suppresses inflammation and protects prostate endothelial cells in animal models.^18^, ^19^ Unfortunately, the exact mechanism of LPS‐induced apoptosis and inflammatory response in H9c2 cells remains unclear.

Adenosine monophosphate‐activated protein kinase (AMPK) as a regulatory protein was involved in cellular energy metabolism and cell apoptosis.^20^, ^21^, ^22^ Activation of AMPK leads to the accumulation of protein p53, which induces cardiomyocyte apoptosis.^23^

Our results revealed that HES attenuated the H9c2 cells' inflammation response and apoptosis induced by LPS by promoting the activation of the AMPK/P53 signaling.

## 2 MATERIALS AND METHODS

### 2.1 Ethical statement

The study was approved and conducted under the ethical committee of Yuhuangding Hospital. This study was approved by the clinical research ethics committee of Yuhuangding Hospital.

### 2.2 H9c2 cell culture

H9c2 cardiac cell lines were cultured in Dulbecco's modified Eagle medium, including 10% (v/v) fetal bovine serum, 100 U/mL penicillin, and 100 mg/mL streptomycin in a humidified atmosphere (5% CO_2_) at 37°C. Cells (2 × 10^6^ cells/well) were seeded onto six‐well plates for 24 h.

### 2.3 Cell treatments

First, HES (Sigma‐Aldrich) was dissolved in dimethyl sulfoxide (DMSO), then HES with different concentrations (5, 10, 15, 20, and 40 µM) was dissolved in the medium at the concentration of LPS (1 µg/mL), and the cells were cultured for 24 h.

### 2.4 AMPK inhibitor

H9c2 cells were exposed to compound C (an AMPK inhibitor, 10 µM), for 30 min before adding HES. Incubation for 24 h, followed by LPS treatment of cells for 24 h. In this way, compound C treatment on LPS‐induced H9c2 cells was evaluated.

### 2.5 MTT assay

H9c2 cells (5 × 10^3^ cells/well) were cultured in 96‐well plates. After incubation with compound C (10 µM) for 1 h and/or HES for 24 h, these cells were then treated with LPS for 24 h. Then, 10 µL MTT solution (5 mg/mL, Sigma‐Aldrich) was added and the plate was incubated at 37°C for an additional 4 h. After centrifugation, the cell culture medium was replaced with DMSO (Sigma‐Aldrich). Finally, the absorbance value in each well of the plate was detected at 570 nm using a plate reader (Bio‐Rad Laboratories, Inc.).

### 2.6 Terminal deoxynucleotidyl transferase‐mediated dUTP nick‐end labeling assay

Apoptosis assay was carried out by terminal deoxynucleotidyl transferase‐mediated dUTP nick‐end labeling (TUNEL) Apoptosis Detection Kit (Green FITC‐labeled Fluorescence Assay, Universal), according to the manufacturer's protocol (Cat: KGA7072, KeyGEN BioTECH). Cells were first allowed to slide, then cells on coverslips were fixed with 4% paraformaldehyde, stained according to TUNEL kit protocol.

### 2.7 Real‐time quantitative PCR

Total RNA was isolated from cultured H9c2 cells using TRIzol reagent (Invitrogen), reverse‐transcribed to cDNA, and analyzed quantitative PCR using CFX96 QPCR System. These primers were as shown: GAPDH, forward: 5′‐GACAT GCCGCCTGGAGAAAC‐3′ and reverse: 5′‐AGCCCAGGATGCCCTTTAGT‐3′; tumor necrosis factor (TNF)‐α, forward: 5′‐AGCATGATCCGAGATGTGGAA‐3′, reverse: 5′‐TAGACAGAA GAGCGTGGTGGC‐3′; IL‐1β, forward: 5′‐GGGATGATGACGACCTGCTA G‐3′ and reverse: 5′‐ACCACTTGTGGCTTATGTTCTG‐3′. GADPH served as the internal control and quantitative measurements were analyzed using the ΔΔCt method.

### 2.8 Western blot assay

H9c2 cells were directly homogenized with Radio Immunoprecipitation Assay Buffer (RIPA, Solaribio), including 1 mM PMSF and protease inhibitor cocktail (Merck). Protein extracts were collected following sample centrifugation. Proteins (60 µg) were then separated in 10% SDS‐PAGE and transferred to a PVDF membrane (Beyotime Institute of Biotechnology). Membranes were then blocked in 5% nonfat dry milk solution for 1 h at room temperature, followed by an overnight incubation at 4°C with primary antibody solution: Bcl‐2 (1:1000; #2870, Cell Signaling Technology), (p)‐AMPK (1:1000; #2535, Cell Signaling Technology), AMPK (1:1000; #5831, Cell Signaling Technology), Bax (1:1000; #2772, Cell Signaling Technology) or rabbit P53 antibody (ab179477; 1:2000; Abcam), and mouse antibodies to β‐actin (1:6000; #3700, Cell Signaling Technology). After washing for 15 min, the membrane was incubated with secondary antibody: anti‐mouse (1:1000; #7076, Cell Signaling Technology)/rabbit (1:1000; #7074, Cell Signaling Technology) antibody, incubated for 1 h at room temperature, then washed three times with TBST for 5 min each, enhanced chemiluminescence solution Color development was performed, and the Western blot analysis system was used to capture the protein image. The gray value of the protein was finally quantified using ImageJ 6.0 (National Institutes of Health).

### 2.9 Statistical analysis

Values are expressed as the mean ± standard deviation (SD). The comparison between the two groups was analyzed through the Student's *t* test. One‐way analysis of variance (ANOVA), with Fisher's least significant difference *t* test, used for comparison between multiple groups. *p* < .05 was considered to have a significant difference.

## 3 RESULTS

### 3.1 HES alleviated cell viability in LPS‐induced H9c2 cells

**Figure 1:**
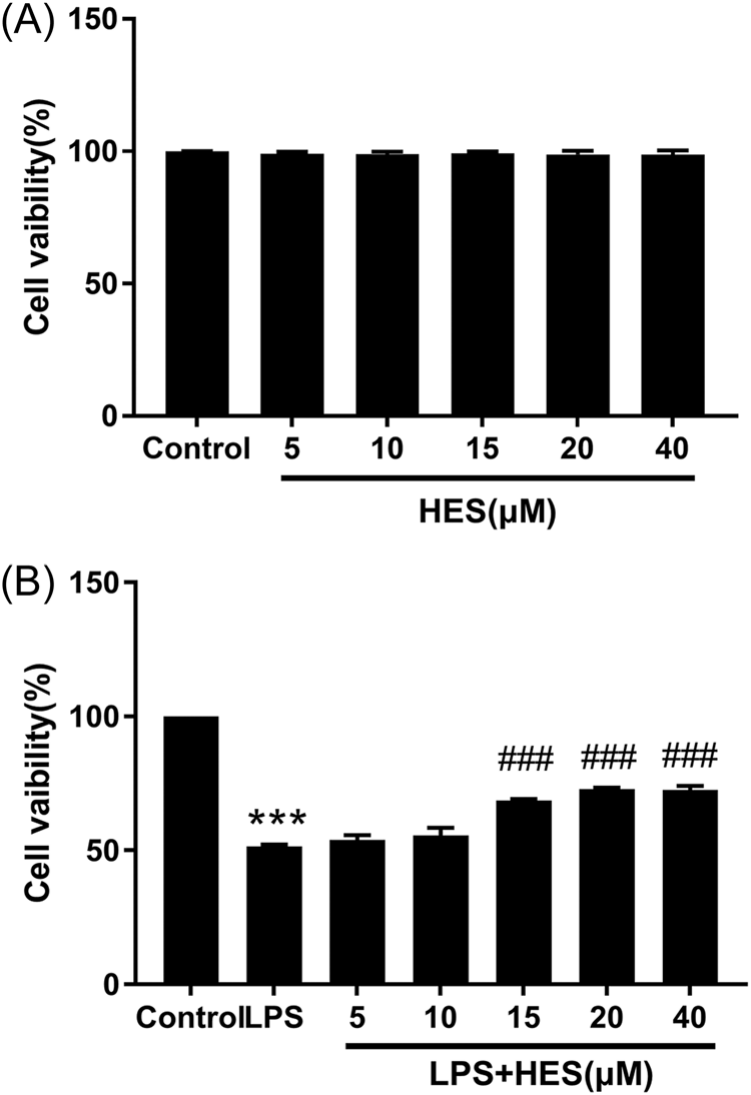
HES alleviated cell viability in LPS‐induced H9c2 cells. (A) H9c2 cells were exposed to the different concentrations of HES (5, 10, 15, 20, and 40 µM) for 24 h. Cell viability was detected by MTT assay. *N* = 3; data were analyzed by one‐way ANOVA. The pairwise comparison after one‐way ANOVA analysis was analyzed by LSD‐t. (B) Viability of H9c2 cells was detected by MTT assay following pretreatment with different concentrations of HES (5, 10, 15, 20, and 40 µM) for 24 h, followed by exposure to 10 µg/mL LPS for 24 h. *N* = 3; data were analyzed by one‐way ANOVA. The pairwise comparison after one‐way ANOVA analysis was analyzed by LSD‐t. ANOVA, analysis of variance; HES, hesperetin; LPS, lipopolysaccharide; LSD‐t, least significant difference *t* test. ***p < .001 versus Control; ^###^ *p* < .001 versus LPS.

H9c2 cells were first exposed to different concentrations (5, 10, 15, 20, and 40 µM) of HES for 24 h, and then we tested the effect of HES on cell viability through MTT experiments. Compared with the control group, there was no significant difference in the survival ability of H9c2 cells when exposed to different concentrations of HES (Figure 1A; *p* > .05). Our results reveal that different concentrations of HES have no significant effect on the viability of H9C2 cells. As shown in Figure 1B, LPS could significantly inhibit H9c2 cells viability compared with the control group (*p* < .001). However, the inhibitory effect of LPS on viability of H9c2 cells was significantly increased by HES in dose‐dependent manner (*p* < .001). Importantly, HES (15 µM) remarkably mitigated LPS‐induced H9c2 cells damage. Overall, these results revealed HES alleviated LPS‐induced H9c2 cells impairment.

### 3.2 HES inhibited LPS‐induced pro‐inflammatory cytokine production in H9c2 cells

**Figure 2:**
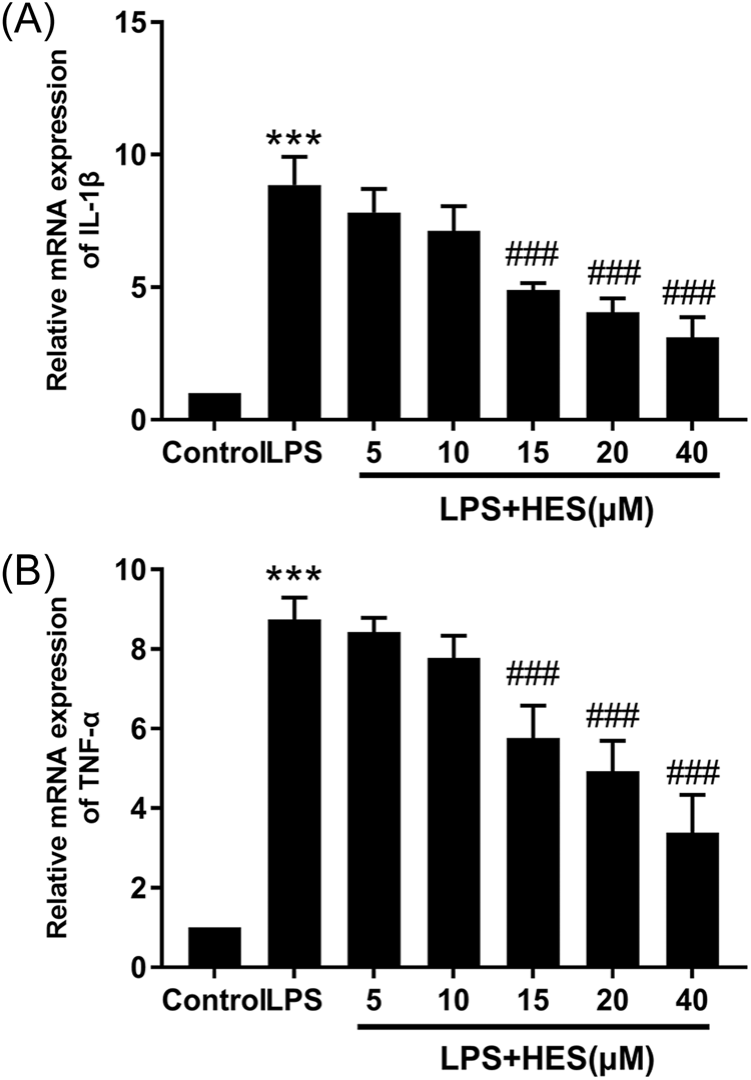
HES inhibited LPS‐induced pro‐inflammatory cytokine production in H9c2 cells. The mRNA expression levels of inflammatory mediators (A) IL‐1β and (B) TNF‐α in H9c2 cardiomyocytes. ***p < .001 versus Control; ^###^ *p* < .001 versus LPS. *N* = 3; Data were analyzed by One‐way ANOVA. The pairwise comparison after one‐way ANOVA analysis was analyzed by LSD‐t. ANOVA, analysis of variance; HES, hesperetin; LPS, lipopolysaccharide; LSD‐t, least significant difference *t* test.

In Figure 2, 24 h after LPS stimulation of H9c2 cardiomyocytes, IL‐1β (Figure 2A, *p* < .001) and TNF‐α (Figure 2B, *p* < .001) mRNA expression were significantly increased compared with the control group. Moreover, different concentrations of HES (5, 10, 15, 20, and 40 µM) were exposed to assess its effect on LPS‐induced IL‐1β and TNF‐α expression. The results demonstrated that HES treatment could significantly attenuate the LPS‐induced elevation of pro‐inflammatory cytokine production compared with the LPS group.

### 3.3 HES attenuated LPS‐induced apoptosis in H9c2 cells

**Figure 3:**
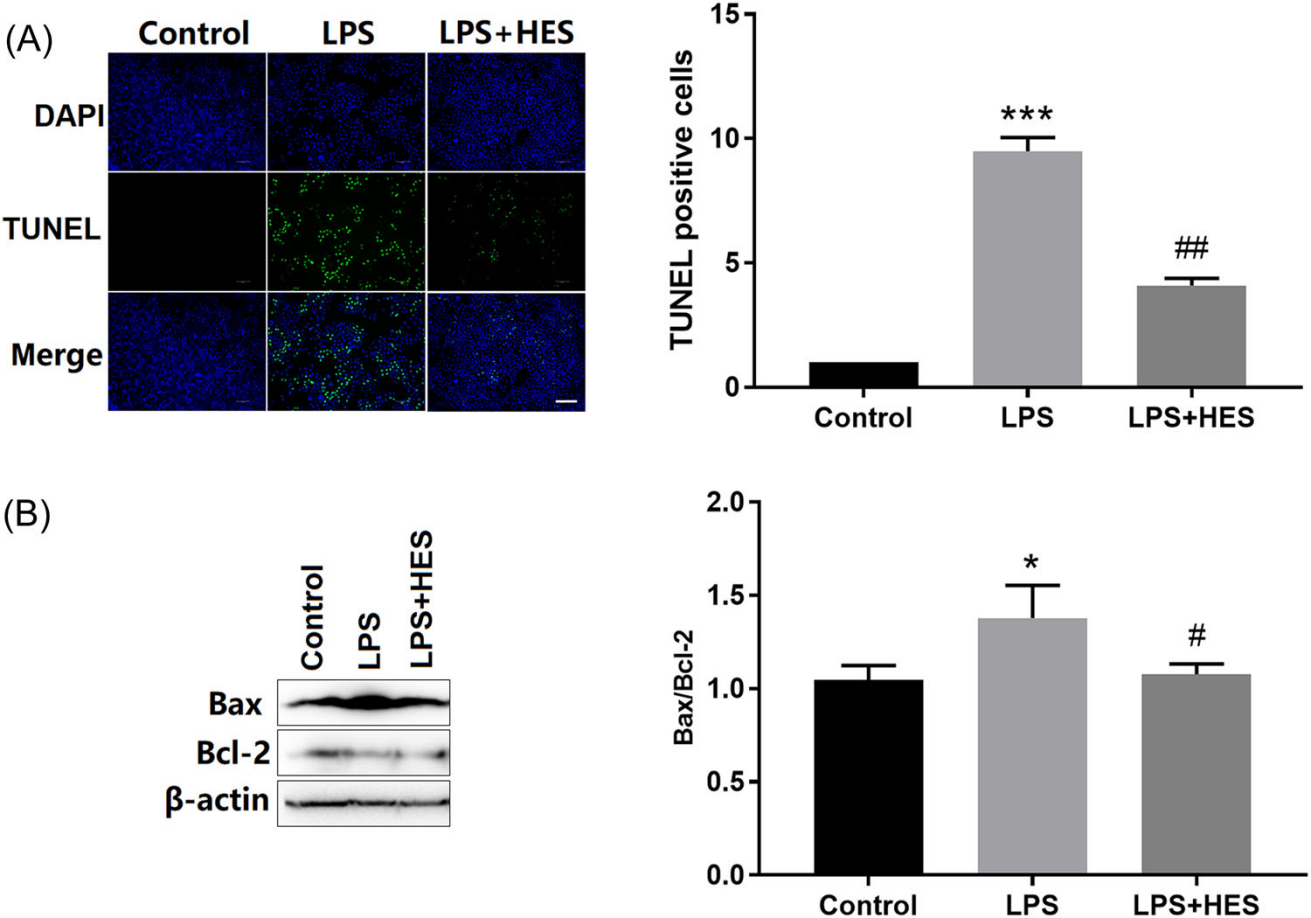
HES attenuated LPS‐induced apoptosis in H9c2 cells. (A) Fluorescence microscopy of H9c2 cardiomyocytes (original magnification, ×400). Quantitative results of TUNEL staining. *N* = 6; data were analyzed by one‐way ANOVA. The pairwise comparison after one‐way ANOVA analysis was analyzed by LSD‐t. ***p < .001 versus Control cells; ^##^ *p* < .01 versus LPS. (B) Representative western blot images (Bax and Bcl‐2). Quantitative results of Bax/Bcl‐2 expression. *N* = 3; data were analyzed by one‐way ANOVA. The pairwise comparison after one‐way ANOVA analysis was analyzed by LSD‐t. ANOVA, analysis of variance; LSD‐t, least significant difference *t* test. *p < .05 versus Control cells; ^#^ *p* < .05 versus LPS.

As shown in Figure 3A, 48 h after LPS stimulation of H9c2 cardiomyocytes, the percentage of TUNEL‐positive nuclei was markedly increased compared with the control cells (*p* < .001). However, HES treatment remarkably reduced the percentage of TUNEL‐positive cells (*p* < .001 vs. the LPS‐only group).

At the same time, we also detected the apoptosis‐related genes protein expression (Figure 3B), such as Bax and Bcl‐2. The result demonstrated that LPS treatment markedly increased the Bax protein expression (*p* < .05 vs. the control), while obviously decreased the Bcl‐2 expression (*p* < .05 vs. the control). However, HES treatment significantly reversed the Bax (*p* < .05 vs. the LPS‐treated cells) and Bcl‐2 protein level (*p* < .05 vs. the LPS‐treated cells). Overall, these results revealed that the HES against LPS‐induced injury may be related to the apoptosis gene expression.

### 3.4 HES upregulates the phosphorylation of AMPK and downregulates the P53 expression in LPS‐induced H9c2 cells

**Figure 4:**
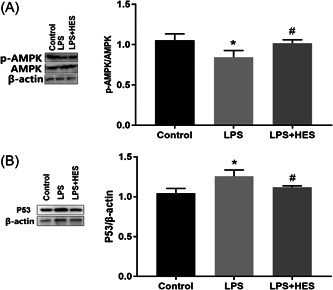
HES upregulates the phosphorylation of AMPK and downregulates the P53 expression in LPS‐induced H9c2 cells. (A) Western blots demonstrate the expression changes of p‐AMPK and AMPK protein. *N* = 3; data were analyzed by One‐way ANOVA. The pairwise comparison after one‐way ANOVA analysis was analyzed by LSD‐t. *p < .05 versus Control cells; ^#^ *p* < .05 versus LPS. (B) Western blots demonstrate the expression changes of P53 protein. *N* = 3; data were analyzed by one‐way ANOVA. The pairwise comparison after one‐way ANOVA analysis was analyzed by LSD‐t. ANOVA, analysis of variance; HES, hesperetin; LPS, lipopolysaccharide; LSD‐t, least significant difference *t* test. *p < .05 versus Control cells; ^#^ *p* < .05 versus LPS.

To further explore the protective mechanism of HES against apoptosis, combined with previous literature, we detected the MAPK/P53 signaling pathway‐related proteins in H9c2 cardiomyocytes treated with LPS. As shown in Figure 4, LPS treatment significantly decreased the phosphorylation of AMPK and increased P53 expression in H9c2 cells. However, HES treatment could reverse this effect in LPS‐induced H9c2 cells. Overall, HES activates the phosphorylation of AMPK and inhibits the P53 expression in LPS‐induced H9c2 cells.

### 3.5 Compound C inhibits the LPS‐mediated cardioprotective effect of HES

**Figure 5:**
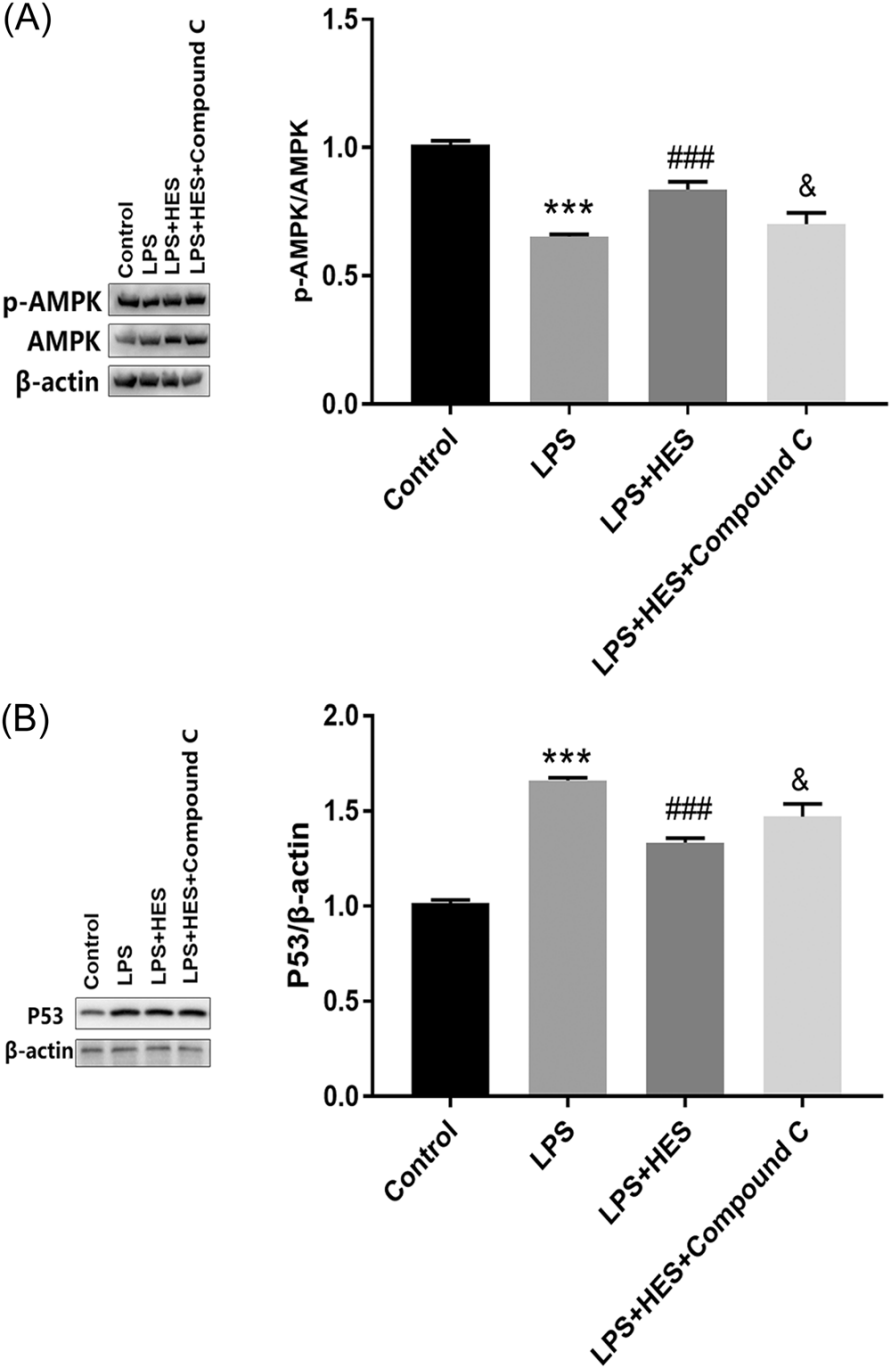
Compound C inhibits the LPS‐mediated cardioprotective effect of HES. (A) AMPK phosphorylation and (B) P53 expression were analyzed by immunoblotting. ***p < .001 versus Control; ^###^ *p* < .001 versus LPS + HES; ^&^ *p* < .05 versus LPS + HES. *N* = 3; data were analyzed by one‐way ANOVA. The pairwise comparison after one‐way ANOVA analysis was analyzed by LSD‐t. ANOVA, analysis of variance; HES, hesperetin; LPS, lipopolysaccharide; LSD‐t, least significant difference *t* test. ***p < .001 versus Control cells; ^###^ *p* < .001 versus LPS; ^&^ *p* < .05 versus LPS + HES.

To investigate the change of the AMPK/P53 pathway in HES protection, H9c2 cells were pretreated with compound C (an AMPK inhibitor), followed by stimulation with LPS and HES. As shown in Figure 5, compound C remarkably weakened the inhibitory effect of HES on the LPS‐induced reduction of AMPK phosphorylation and elevation of P53 expression. Overall, our findings revealed that compound C inhibits the LPS‐mediated cardioprotective effect of HES.

## 4 DISCUSSION

In our study, LPS treatment resulted in inflammatory and apoptotic responses in H9c2 cardiomyocytes, whereas HES markedly attenuated the inflammatory responses in H9c2 cardiomyocytes by inhibiting the pro‐inflammatory cytokines IL‐1β and TNF‐α expression. Moreover, HES has a protective effect on LPS‐induced H9c2 cardiomyocyte apoptosis. Finally, HES inhibits the injury response of cardiomyocytes by regulating the AMPK/P53 signaling pathway.

Myocardial inflammation has been reported in many previous studies.^24^, ^25^, ^26^ In addition, reductions in cardiac inflammation may have beneficial effects on cardiac dysfunction.^27^ LPS‐induced cardiomyocyte inflammatory responses are characterized by inflammatory mediators.^28^ In this study, HES treatment downregulated LPS‐induced inflammatory factor expression, suggesting that HES may protect myocardial cells from inflammatory reaction to some extent.

A previous study has reported that the number of inflammatory responses and apoptosis coexisted in cardiomyocytes in the LPS‐treated mouse model of sepsis.^29^ Meanwhile, whether cells undergo apoptosis depends on the balance between the expression of common proapoptotic proteins and antiapoptotic proteins.^30^, ^31^ Our results reveal that HES can counteract LPS‐induced cell apoptosis by inhibiting the expression of proapoptotic protein Bax and increasing the expression of apoptotic Bcl‐2.

Previous studies have reported that AMPK can inhibit the proliferation of nonmalignant cells.^32^ Meanwhile, P53 serves as an important regulatory factor related to cell cycle arrest and apoptosis in tumor cells.^33^ Moreover, the AMPK/P53 pathway has also been proven to play a crucial role in DOX‐induced cardiomyocyte death.^34^, ^35^ Therefore, in our study, in H9c2 cells treated with LPS, the phosphorylation level of AMPK protein was significantly downregulated, and the expression of P53 protein was significantly increased, while HES treatment reversed the expression of these proteins. In addition, the expression of the proapoptotic gene Bax was significantly increased in H9c2 cells treated with LPS, while the expression of Bcl‐2 was significantly reduced. In conclusion, the above results confirm that the AMPK/P53 pathway is involved in LPS‐induced cardiomyocyte apoptosis.

To further investigate the function of HES in cardioprotection, the effect of LPS treatment on the AMPK/P53 pathway in H9c2 cells was examined. These results showed that HES obviously attenuated the LPS‐induced increase in AMPK phosphorylation level and P53 expression. In addition, AMPK, as a sensor and regulator of cellular energy, can regulate cell apoptosis and cell proliferative capacity to maintain cellular homeostasis. Therefore, our study confirmed that HES attenuated LPS‐induced apoptosis of H9c2 cardiomyocytes, thereby activating the expression of AMPK and p53 proteins. Meanwhile, the AMPK phosphorylation inhibitor compound C was applied to inhibit the phosphorylation level of AMPK, and the results showed that the effects of HES on H9c2 cell apoptosis, AMPK phosphorylation, and P53 expression were reversed. Taken together, these results suggest that HES prevents LPS‐induced cardiomyocyte injury by activating the AMPK/P53 signaling pathway.

In conclusion, our study revealed that HES could protect H9c2 cardiomyocytes from LPS‐induced inflammation and apoptosis by activating the AMPK/P53 signaling pathway, which provides a certain scientific basis for the prevention and treatment of LPS‐induced myocardial injury.

There are also some limitations to our research, for example, (1) the main components of HES that play a role in myocarditis are not clear. (2) Lack of animal myocardial model experiments using HES gavage treatment.

## AUTHOR CONTRIBUTIONS

**Zan Xie**: Investigation; methodology; writing—original draft; writing—review and editing. **Chunxia Yang**: Investigation; methodology; writing—original draft. **Tingting Xu**: Conceptualization; data curation; formal analysis; funding acquisition; project administration; supervision; validation; writing—original draft; writing—review and editing.

## CONFLICT OF INTEREST STATEMENT

The authors declare no conflict of interest.
